# Pituitary Imaging Abnormalities and Related Endocrine Disorders in Erdheim–Chester Disease

**DOI:** 10.3390/cancers13164126

**Published:** 2021-08-17

**Authors:** Skand Shekhar, Jorge A. Irizarry-Caro, Ninet Sinaii, William A. Gahl, Juvianee I. Estrada-Veras, Rahul H. Dave, Bernadette R. Gochuico, Georgios Z. Papadakis, Nicholas Patronas, Constantine A. Stratakis, Kevin O’Brien, Fady Hannah-Shmouni

**Affiliations:** 1 Reproductive Physiology and Pathophysiology Group, Clinical Research Branch, National Institute of Environmental Health Sciences, NIH, Bethesda, MD 20892, USA; skand.shekhar@nih.gov; 2 Section on Genetics and Endocrinology, Eunice Kennedy Shriver National Institute of Child Health and Human Development, NIH, Bethesda, MD 20892, USA; stratakc@cc1.nichd.nih.gov; 3 Office of the Clinical Director, National Human Genome Research Institute, NIH, Bethesda, MD 20892, USA; Jorge.a.irizarrycaro@uth.tmc.edu (J.A.I.-C.); gahlw@nih.gov (W.A.G.); juvianee.estradaveras@nih.gov (J.I.E.-V.); gochuicb@mail.nih.gov (B.R.G.); obrienke@mail.nih.gov (K.O.); 4 Department of Internal Medicine, University of Texas Health Science Center at Houston, Houston, TX 77030, USA; 5 Clinical Center, NIH, Bethesda, MD 20892, USA; sinaiin@cc.nih.gov (N.S.); njpatronas@gmail.com (N.P.); 6 Department of Neurology, Inova Fairfax- University of Virginia College of Medicine, Fairfax, VA 22031, USA; rahul.dave@inova.org; 7 Department of Radiology, Medical School, University of Crete, 71500 Heraklion, Greece; papadakisg@ics.forth.gr; 8 Foundation for Research and Technology Hellas, Computational Biomedicine Laboratory, 71110 Heraklion, Greece

**Keywords:** Erdheim–Chester disease, pituitary, hypothalamus, hypopituitarism, diabetes insipidus, histiocytosis, endocrinology

## Abstract

**Simple Summary:**

Erdheim–Chester disease (ECD) is a rare histiocytic neoplasm that is frequently associated with hypothalamic–pituitary gland involvement leading to endocrine dysfunctions. Frequently, endocrinopathy is permanent and precedes the diagnosis of ECD and may also develop during the course of treatment. However, the exact nature and frequency of hypothalamic–pituitary involvement are unknown. We studied a natural history cohort of 61 subjects with Erdheim–Chester disease and found abnormal pituitary imaging in 47.5% of cases, associated with panhypopituitarism, diabetes insipidus, and a higher median number of pituitary endocrinopathies. These data confirm and significantly extend previous reports of centrally occurring endocrine dysfunction and highlight the need for routine imaging and systematic assessment of hypothalamic–pituitary endocrine function in patients with ECD.

**Abstract:**

Purpose: We examined abnormal pituitary imaging (API) and associated endocrine dysfunction in subjects with ECD. Methods: A cross-sectional descriptive examination of a natural history cohort study diagnosed with ECD was conducted at a clinical research center. Subjects underwent baseline endocrine tests of anterior and posterior pituitary function and dedicated pituitary gland MRI scans. We determined the frequency of various pituitary imaging abnormalities in ECD and assessed its relationships with age, sex, body mass index (BMI), *BRAF* V600E status, high sensitivity C-reactive protein (hsCRP), erythrocyte sedimentation rate (ESR), pituitary hormone deficits and number, diabetes insipidus (DI), and panhypopituitarism. Results: Our cohort included 61 subjects with ECD [age (SD): 54.3 (10.9) y, 46 males/15 females]. API was present in 47.5% (29/61) of ECD subjects. Loss of the posterior pituitary bright spot (36.1%) followed by thickened pituitary stalk (24.6%), abnormal enhancement (18.0%), and pituitary atrophy (14.8%) were the most common abnormalities. DI and panhypopituitarism were more frequent in subjects with API without differences in age, sex distribution, hsCRP, ESR, and *BRAF* V600E status compared to normal pituitary imaging. Conclusions: We noted a high burden of API and endocrinopathies in ECD. API was highly associated with the presence of panhypopituitarism and DI. Therefore, a thorough assessment of hypothalamic–pituitary integrity should be considered in subjects with ECD.

## 1. Introduction

Erdheim Chester disease (ECD), a non-Langerhans histiocytosis, was initially described by Erdheim and Chester as lipoid granulomatosis in 1930 [1]; it was recently reclassified by the WHO as a neoplasm of histiocytic origin [2]. ECD is extremely rare and has been reported a few hundred times in the literature with a mean presenting age of ~53 years, predominantly affecting males [2,3]. Disruptions in molecular genetic pathways such as *BRAF* V600E and MAP kinase in a group of hematopoietic cells lead to their increased production and prolonged survival, which is implicated in the causation of ECD [4,5,6]. Additionally, *NRAS*, *KRAS*, *PIK3CA*, *ARAF*, and *MAP2K1* defects have been involved in the pathogenesis of ECD [7,8]. Initial clinical manifestations of ECD include ostalgia (50%), neurological symptoms (23%), and diabetes insipidus (DI, 22%), which is the most frequently overlooked symptom [3]. We have also previously noted that ECD leads to a wide variety of endocrinopathies such as hypothyroidism, adrenal dysfunction, and hypogonadism [9,10,11,12,13]. Additionally, ECD can also affect the retroperitoneum, pulmonary, cardiovascular, and cutaneous systems [12,14,15,16,17].

Treatment is usually warranted for subjects with symptoms, particularly those with neurological abnormalities or other end-organ damage [18]. In resource-rich settings, the current mainstay of therapy consists of targeted therapies such as *BRAF* V600E inhibitors and MEK inhibitors as appropriate [18,19,20]. Interferon alpha is a reasonable treatment alternative in resource-limited settings, whereas glucocorticoid use has fallen out of favor in the treatment of ECD.

Endocrinopathies in ECD occur primarily through the histiocytic involvement of the hypothalamus, infundibular stalk, and pituitary gland. These may manifest as abnormal pituitary imaging (API), with or without biochemical abnormalities, but their frequency and characteristics have not been extensively studied. One retrospective study reported a significant proportion of API (26.8%) in a subgroup of ECD subjects that had pituitary imaging [17]. Further, the association of API with sex, age, BMI, hypothalamic–pituitary hormone dysfunction, *BRAF* V600E status, and inflammation (hs-CRP levels) is unknown. Hence, we performed a cross-sectional analysis of a cohort of biopsy-proven ECD subjects enrolled in a large natural history study to determine the frequency of API and its association with pituitary endocrinopathies and other demographic and disease-related factors.

## 2. Methods

### 2.1. Overview and Objectives

The present study is a cross-sectional analysis of a larger National Institutes of Health IRB-approved natural history study “Clinical and Basic Investigations into Erdheim–Chester disease” (Protocol 11-HG-0207, ClinicalTrials.gov Identifier: NCT01417520). Clinical, biochemical, and radiological features of a cohort of biopsy-proven ECD subjects were recorded between 2011 and 2018. Informed consent was obtained from all participants, after which the history was obtained and physical examination and investigations were performed, as published previously [12]. The diagnosis of ECD was based on the ECD consensus criteria [21].

### 2.2. Procedures

Biopsy specimens for all enrolled subjects were reviewed by a pathologist who verified the diagnosis of ECD. All subjects underwent screening for *BRAF* V600E variants. Those not harboring a *BRAF* V600E pathogenic variant were tested for *KRAS*, *NRAS*, *MAP2K1*, *PIK3CA*, and *ARAF* (MAP Kinase) gene variants. At the time of enrollment, some subjects were being treated with *BRAF* modulators or interferon therapy (Supplement 1). MRI of the pituitary was performed in 56 subjects and the remaining five had CT scans of the sella. MRI examination was performed on a 3.0 Tesla Philips Achieva^®^ using T1 and T2-weighted imaging with sagittal, coronal, and axial sequences. Unenhanced and contrast enhanced images were obtained before and after intravenous injection of Magnevist^®^ (gadopentetate dimeglumine) contrast. Special thin tomographic sections were obtained in the orbits and in the pituitary gland. Fat suppression techniques were used in the orbits. For those who had CT scans, the examination was performed before and after intravenous administration of contrast material (Isovue). Axial images were obtained at 2 mm intervals. The scans were reviewed and reported by a certified NIH neuroradiologist and confirmed by neuroendocrinologists (FHS, SS) with expertise in pituitary disorders. We categorized all imaging abnormalities that could be attributed to ECD involvement, such as empty sella, pituitary stalk thickening, loss of posterior bright spot, pituitary atrophy, and abnormal enhancement of the pituitary, as API. Pituitary stalk thickness was measured at the level of the median eminence and at the midpoint between the median eminence and the dorsum sellae (insertion into the pituitary gland) in the coronal sections. Consistent with previous reports, a median eminence-level stalk thickness of ≥4 mm or a midpoint-level stalk thickness of ≥3 mm was classified as pituitary stalk thickening [22]. A significant reduction in pituitary size (maximum vertical dimension ≤ 2 mm) but with identifiable pituitary tissue was classified as partly empty sella; when the largest vertical dimension of the pituitary was at or just below the physiological limit of ~4 mm, we labelled it pituitary atrophy. Abnormal pituitary enhancement was defined as heterogenous appearance of the entire pituitary gland parenchyma on T1-weighted post-contrast MRI images or post contrast CT scans [23]. Incidental findings such as physiological variation in pituitary dimensions, Rathke’s cyst or pars intermedia cyst were categorized as being unrelated to ECD and thus excluded for our analysis of API in this study. We classified a scan as abnormal pituitary imaging if more than one imaging abnormality was present and authors determined that the findings were likely related to pituitary involvement by ECD. Any isolated imaging defect by itself did not constitute API. For example, an isolated small pituitary or an isolated stalk deviation or an isolated pituitary pars intermedia cyst may represent incidental findings and for the purpose of our study did not constitute API.

Panhypopituitarism and number of pituitary deficits (centrally occurring hypothyroidism, hypogonadism, DI, and adrenal insufficiency [AI]) were diagnosed by an endocrinologist based on the pituitary hormonal evaluation as outlined by the Endocrine Society guidelines [24]. Specifically, we performed serum free T4 and TSH to test for the hypothalamic–pituitary–thyroid axis, and any low normal or subnormal free T4 combined with low or inappropriately normal TSH was classified as central hypothyroidism [9,10,25]. Central hypogonadism was diagnosed on the basis of two morning serum total testosterone values below 300 ng/dL (or below our laboratory reference range) and low or inappropriately normal serum LH and FSH in association with hypogonadal symptoms. Central adrenal insufficiency was diagnosed in those patients a) who were on supraphysiological glucocorticoid replacement (prednisone equivalent dose > 5 mg/day) for more than two weeks or had b) a morning cortisol of <5 µg/dL in combination with ACTH as a preliminary test. A cosyntropin stimulation test was performed if clinically or biochemically indicated to confirm the diagnosis [11,13]. All patients were screened for DI with a clinical history followed by serum sodium (and electrolytes) and 24-h urine volume and osmolarity. Diabetes insipidus was diagnosed if patients were on vasopressin when enrolled in the study or had classic symptoms and biochemical findings (e.g., elevated serum sodium with low urinary osmolality) suggestive of DI [12]. We did not perform evaluation for growth hormone deficiency with serum GH or IGF-1 levels. Furthermore, neurological screening was performed by experienced neurologists using appropriate clinical and imaging assessments listed in detail elsewhere [26]. Cognitive impairment, neuropathies, and cranial nerve involvement were classified as ‘neurodegeneration/cognitive impairment’ while coordination impairment and pyramidal tract involvement were classified as ‘cerebellar syndrome’ due to their distinct clinical features [26]. Overall, neurological abnormalities reflect any neurological involvement by ECD including ‘neurodegeneration/cognitive impairment’ and/or ‘cerebellar syndrome’.

### 2.3. Statistical Analysis

Data were assessed for distributional assumptions, and continuous data were compared by the two-sample Student *t*-test or non-parametric Wilcoxon rank-sum tests, as appropriate. Fischer’s exact test was used for comparing categorical data. Exact 95% confidence intervals (CI) were computed from binomial proportions. Data were analyzed using SAS v9.4 (SAS Institute, Inc., Cary, NC, USA). Statistical evidence was based on the size and magnitude of the effects, along with *p*-values.

## 3. Results

We included sixty-one subjects who were enrolled in the ECD cohort with a mean age (SD) of 54.3 (±10.9) years. A majority were males (*n* = 46, 75.4%), and the mean duration of diagnosis of 4.2 years (Table 1). Subjects presented to the NIH at a mean of 2.9 ± 0.4 years after they were formally diagnosed with ECD and at a mean of 6.6 ± 0.8 years after first signs of ECD had appeared. Pituitary imaging was performed on their initial enrollment visit. Out of the 61 patients with ECD, 57 had interpretable *BRAF* sequencing performed (four lacked tissue for examination) and 31 were found to have a pathogenic variant in *BRAF* V600E while none of them were germline. Among the remaining subjects, *ARAF* D228V pathogenic variant and an activating *ALK* fusion (KIF5B-ALK) were found in one subject each. Further details are reported elsewhere [12]. Pituitary imaging was performed on their initial enrollment visit.

API was present in 47.5% (29/61; 95% CI 34.6–60.7%) of subjects. Ten subjects (out of 61) who had normal baseline pituitary scans had follow-up MRI scans (median: 2 years; range: 1–6 years) and continued to show normal findings.

### 3.1. Types of Abnormal Pituitary Imaging

**Figure 1:**
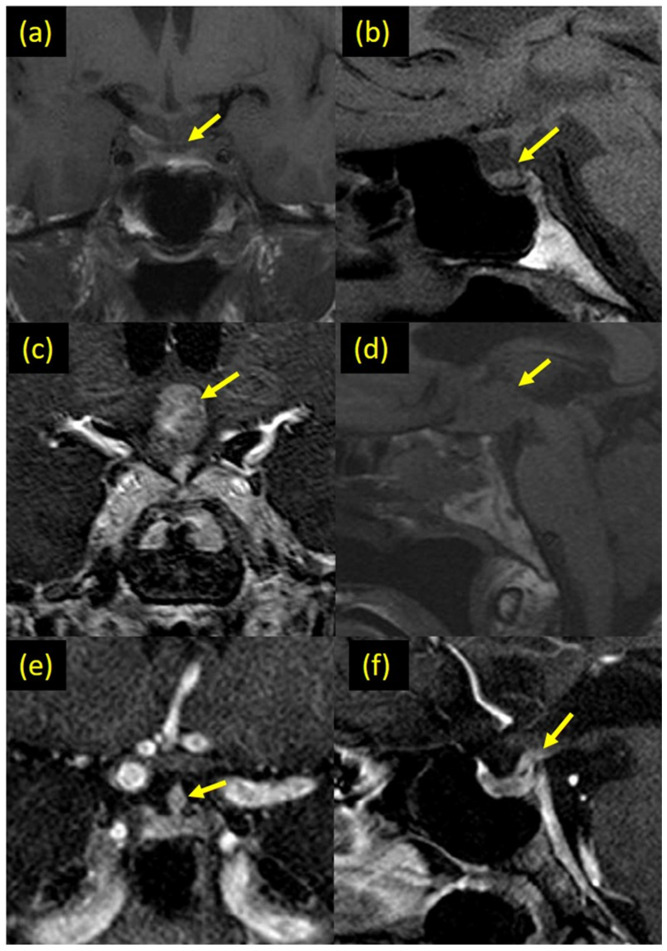
(**a**,**b**) T1 weighted MRI images of the skull base showing a small size pituitary gland with significant narrowing of the pituitary stalk. (**c**) Coronal T1-weighted post-contrast and (**d**) sagittal T1-weighted pre-contrast MR-images showing suprasellar post-chiasmatic mass involving the pituitary stalk and the hypothalamic floor, which is intensely enhanced in the post-contrast images. (**e**) Coronal T1-weighted post-contrast MR-image showing focal engorgement of the middle segment of the pituitary stalk due to ECD infiltration (early ECD involvement of the pituitary stalk. (**f**) Sagittal T1-weighted post-contrast MR-image showing abnormal thickening of the most proximal end of the pituitary stalk, at the point of its insertion into the hypothalamic floor.

The most common pituitary imaging abnormality was the loss of the posterior pituitary bright spot (36.1%) followed by a thickened pituitary stalk (24.6%), abnormal pituitary gland enhancement (18.0%), pituitary atrophy (14.8%), and stalk deviation (11.5%). Pituitary encasement (4.9%) and microadenoma (1.6%) were less common. There was no invasion of the cavernous sinuses and suprasellar cistern. Incidental pituitary imaging findings included pars intermedia cysts (4.9%) and Rathke’s cysts (1.6%) (Table 2). Not every subject with a pituitary imaging finding was considered to have API because we did not consider isolated, possibly incidental findings as API. Therefore, while 37 subjects had some pituitary imaging findings, only 29 were considered to have API. The remaining eight subjects had an isolated small pituitary (*n* = 3), an isolated pituitary stalk deviation without thickening (*n* = 1), a Rathke’s cyst (*n* = 1), and a Pars Intermedia cyst (*n* = 3). Individual endocrine, pituitary imaging and treatment characteristics are listed in supplemental Table S1. Figure 1 depicts representative pituitary imaging abnormalities of subjects with ECD. Supplement 2 contains pituitary scans of all subjects with API.

### 3.2. Types of Pituitary Endocrinopathies

**Figure 2:**
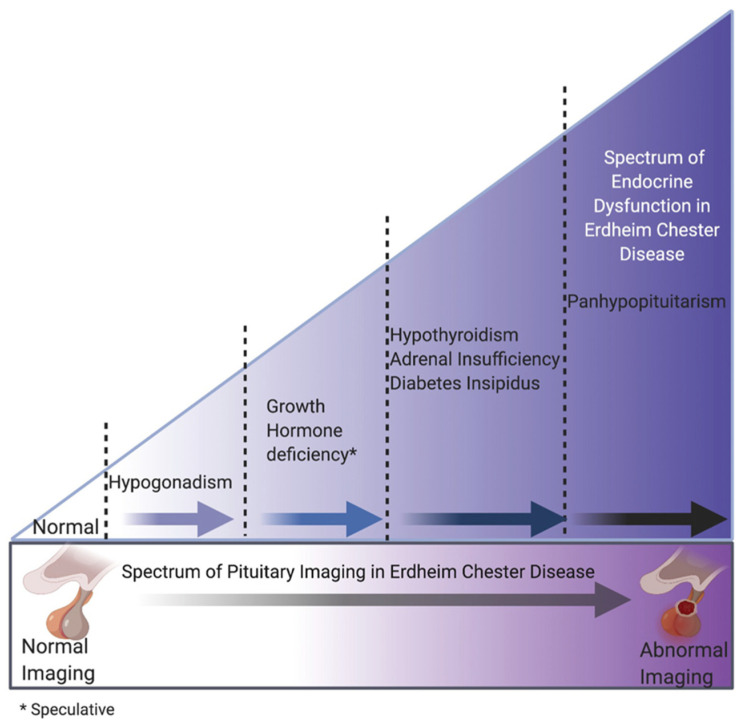
Proposed progression of endocrine and radiological pituitary abnormalities. Note that all hormone deficits listed are centrally occurring and hypogonadism may occur secondary to hyperprolactinemia.

Hypothalamic–pituitary endocrinopathies in ECD were present in 65.5% of subjects. Central hypogonadism was the most frequent endocrine derangement in males (52.46%) followed by central DI (36.05%), central adrenal insufficiency (21.31%), hyperprolactinemia (13.11%), and central hypothyroidism (9.83%). While we did not systematically test for growth hormone deficiency or perform dynamic endocrine testing, panhypopituitarism (defined as ≥3 pituitary hormonal deficiencies) was present in 14.75% of cases. We did not include hyperprolactinemia in analyzing panhypopituitarism due to its likely source being infundibular involvement as opposed to a prolactinoma (‘stalk effect’) [27,28]. Given the frequency of hormone deficits, associations with API and prior knowledge of progressive pituitary endocrinopathies from other etiologies, we propose a schematic model of endocrine and imaging abnormalities in ECD. Figure 2 presents a representative endocrine–radiological schema based on our findings.

### 3.3. Factors Associated with Abnormal Pituitary Imaging in Subjects with ECD

Subjects with API were younger [50.6 ± 11.7 y (95%CI: 46.09–55.01) vs. 57.7 ± 9.0 y (54.42–60.95); *p* = 0.010] but there were no differences in the proportion of females, BMI, *BRAF* V600E pathogenic variants, ESR, and hsCRP between those with abnormal and normal pituitary imaging (Table 1).

Disproportionately higher rates of DI [81.8% vs. 18.2%, difference 63.6%, 95% CI 44.3–83.0%; *p* < 0.001], panhypopituitarism [100.0% vs. 0%; *p* < 0.001], and more pituitary deficits [median (IQR): 2.0 (1.0-3.0) vs. 1.0 (0–1.0); *p* = 0.004)] were observed in subjects with API compared to those with seemingly normal pituitary imaging (Table 1). However, there was no association between API and central adrenal insufficiency, central hypothyroidism, and central hypogonadism (Table 1). Furthermore, while a thickened pituitary stalk was not associated with the presence of DI [46.7% vs. 32.6%, difference 14.1%, 95% CI −14.6–42.7%; *p* = 0.36], loss of the posterior bright spot (on unenhanced T1 weighted imaging) was associated with the presence of DI [68.2% vs. 18.4%, difference 49.8%, 95% CI 26.7–72.8%; *p* = 0.0002]. There was no association between API and inflammatory marker ESR compared to normal pituitary imaging [28.0 (12–49) vs. 21.0 (9.5–26.5) mm/h; *p* =0.079]. Another inflammatory marker, hsCRP, had similar values for the two imaging categories (Table 1). Furthermore, there was no association between API and (1) cerebellar syndrome [no API vs. API: 50% vs. 50%, OR 1.20 (95%CI 0.43–3.29); *p* = 0.79], (2) neurodegeneration/cognitive impairment [no API vs. API: 50% vs. 50%, OR: 1.21 (95%CI 0.44–3.32); *p* = 0.79] or (3) or overall neurological impairment [no API vs. API: 51.1% vs. 48.9%, OR: 1.23 (95%CI 0.39–3.87); *p* = 0.78].

## 4. Discussion

We performed a large scale, cross-sectional descriptive study of a cohort of ECD subjects and found high rates of API. API was not associated with female sex, BMI, age, *BRAF* V600E pathogenic variant or hsCRP. However, subjects with API tended to have higher ESR levels and had a significantly higher number of pituitary deficits than those with normal imaging. We also noted higher rates of DI and panhypopituitarism in those with API. However, we did not find a significant association between API and central AI, central hypothyroidism, and central hypogonadism. These results indicate that more advanced pituitary dysfunctions such as panhypopituitarism are associated with visible deformities of pituitary imaging. Our results also suggest that younger subjects with a higher number of pituitary endocrinopathies and those with DI had a higher prevalence of API.

Community estimates of the frequency of API, most commonly involving pituitary adenomas, range from 9.3–10% [29,30]. Kuo et al. (2021) assessed pituitary imaging in 3840 individuals without known or suspected pituitary disease over one year and found that the frequency of API was ~1.2%, with partially or completely empty sella being the most common incidentalomas [31], indicating an even lower prevalence of pituitary imaging lesions in the community. Authors from the same institution had previously reported a 10.8% prevalence of pituitary imaging abnormalities in those who underwent pituitary imaging for symptoms such as visual or auditory abnormalities that may represent symptomatic pituitary lesions [32]. These estimates are lower than those of our ECD cohort (47.5%). Loss of the posterior pituitary bright spot, thickened pituitary stalk, pituitary atrophy, and abnormal enhancement of the pituitary were the most frequent pituitary imaging abnormalities in our study (Table 2). Similar to Courtillot et al. (2016), loss of the posterior pituitary bright spot in T1 imaging was the commonest pituitary imaging finding in our study population (36.1%) but was less frequent than in their study population (60%). Furthermore, we found a much higher percentage of stalk thickening (24.6%) compared to Courtillot et al. (2016) (13.1%) and also found a higher absolute frequency of pituitary imaging abnormalities than reported by that group (47.5% vs. 24.4%) [17]. Moreover, their study did not report data on pituitary imaging lesions such as suprasellar masses, stalk deviation, empty sella, microadenoma, and pituitary endocrine-imaging associations [17].

Previous smaller studies have reported hypothalamic–pituitary imaging abnormalities in ECD but did not provide data on their nature and impact on endocrine functions. Kumar et al. (2018) reported involvement of the posterior lobe of the pituitary and the infundibulum, but the exact frequency of involvement was not detected [33]. Similarly, Veyssier-Belot et al. (1996) reported DI as a frequent finding in their study population of 59 ECD subjects but did not provide data on pituitary imaging characteristics. [34] Another study of 33 ECD subjects noted a 53% prevalence of abnormal hypothalamic–pituitary imaging with high frequencies of pituitary stalk thickening and loss of the T1 neurohypophyseal bright spot [35]. Abnormalities of orbital and meningeal regions were also frequent in their study [35]. Sedrak et al., in a series of 11 subjects, reported API in 36%, all of whom had DI [36]. In comparison, API was common but not universal in our ECD subjects with DI (~82% had abnormal imaging). Imaging abnormalities in the Sedrak study population included an enlarged pituitary stalk, pituitary involvement, and hypothalamic FLAIR hyperintensity [36]. A review of 60 cases reported involvement of the hypothalamic–pituitary region as a common abnormality, but the frequency and relationship to endocrine disorders such as DI (29% in their series) or panhypopituitarism were not characterized [37]. The authors concluded that the frequency of neuro-imaging abnormalities was at least 30% and remained underdiagnosed [37]. Differences in the frequency of various imaging findings between us and previous studies may be related to differences in study designs such as independent reviews of pituitary scans by a specialized neuroradiologist and two neuroendocrinologists in our study. Other reasons for differences may include enrollment of subjects at different stages of ECD that influenced the timing of pituitary scans or differences in sensitivity of pituitary scans (slice thickness, tesla value of the MRI machine, etc.). Furthermore, except for Courtillot et al. (2016), no prior study has performed a systematic assessment of endocrine function in a comparable cohort and thus we cannot compare estimates of endocrine dysfunction [17].

We did not find any sex, age or BMI differences in those with API. Furthermore, we found no association between inflammatory marker ESR and hsCRP, consistent with similar findings in hypothyroidism [9]. In contrast, previous findings suggested that increased systemic inflammation may accompany adrenal gland involvement [11]. Furthermore, we found that a higher number of pituitary hormone deficits and a higher frequency of panhypopituitarism in subjects with API, suggesting that endocrine dysfunction precedes radiological pituitary abnormalities in ECD and may occur along a spectrum. To represent this spectrum, we propose a schematic model (Figure 2). Moreover, in subjects with hyperprolactinemia, we found only modest elevations [38] in a majority, suggesting that elevated prolactin was likely due to stalk infiltration and reduced dopaminergic signaling rather than a pathologic pituitary process such as a prolactinoma [27,28]. Central hypogonadism was also frequently seen in our study population (47.54%). While histiocytic infiltration of hypothalamic neurons involved in GnRH, LH, and FSH interplay is one plausible reason, chronic systemic inflammation associated with ECD also likely contributes to the high prevalence of hypogonadism. Thus, the overall high frequency of hypogonadism is likely to be multifactorial in our study population. Additionally, DI was frequently associated with API and loss of the physiological posterior bright spot, suggesting that DI may occur in the presence of early imaging abnormalities such as posterior bright spot loss without visible stalk involvement. On the other hand, the frequency of pathogenic variants in *BRAF* V600E was similar in subjects with normal and API, indicating that pituitary abnormalities could not be predicted by molecular characteristics which is contrast to the involvement of other endocrine organs such as the adrenal glands [11,13].

Central nervous system involvement in ECD remains a major cause of morbidity and mortality [26]. For instance, a survival analysis of 53 cases of ECD reported CNS involvement in 51%; these were directly responsible for 29% of reported deaths [18]. Additionally, CNS involvement was an independent, poor prognostic factor for mortality in ECD subjects at five years in their study [18]. Neurologic symptoms can be the presenting feature of ECD, and given the mean delay in ECD diagnosis of 4.2 years, ECD must be considered in patients with inflammatory, infectious or neoplastic-appearing white matter [26]. Clinicians treating ECD should be aware of the high prevalence of API and the association between CNS lesions and mortality. It is imperative that ECD subjects with or without API undergo comprehensive endocrine evaluation and be treated in accordance with standard guidelines [24]. Importantly, the diagnosis of central DI and other pituitary endocrinopathies may precede the diagnosis of ECD by several years and may develop during its treatment and persist thereafter [21]. Therefore, we suggest performing an annual pituitary panel [serum prolactin, ACTH, morning cortisol, TSH, free T4, GH, IGF-1, LH, FSH, estradiol (females), morning total testosterone (males)], serum, and urine electrolytes (Na^+^, K^+^) and urinary osmolarity in all patients diagnosed with ECD, in agreement with ECD consensus guidelines [21,39]. Biochemical testing should be accompanied by a baseline enhanced pituitary MRI (primary diagnostic modality) followed by serial scans as clinically indicated [39]. In ECD, proper clinical, biochemical, radiographic, and genetic studies may obviate the need for surgical biopsy or resection of the hypothalamic–pituitary gland.

Our study had some limitations. First, a majority but not all subjects were screened with a pituitary MRI due to logistical or patient-related constraints. Second, we were not able to evaluate the longitudinal effects of treatment on pituitary imaging abnormalities. Third, we could not estimate the incidence of API and endocrinopathies (and their exact frequency) since this was a cross-sectional study. Fourth, we did not perform dynamic endocrine testing to ascertain each endocrinopathy in ECD subjects due to the limitations of our research protocol. This implies that patients who did not have overt symptoms of adrenal insufficiency and had normal morning cortisol, i.e., 5–15 μg/dL, did not have dynamic cosyntropin stimulation testing that may have led to underestimation of the prevalence of central AI. Fifth, we did not test for pathogenic variants in genes other than *BRAF* V600E. Finally, at the time of the NIH evaluation, the mean duration of diagnosis was relatively short (4.2 years), and we may not have captured the exact frequency of API and endocrinopathies.

## 5. Conclusions

To date, our study is the most comprehensive review of API associated with pituitary hormonal dysfunction. This work confirmed a high frequency of API in subjects with ECD and the need for a comprehensive endocrine evaluation. Clinicians should carefully and serially evaluate this at risk population for pre-existing or new-onset endocrine derangements. In subjects with API, panhypopituitarism and DI are more common and thus require close monitoring. More studies should focus on the effects of treatment on API and the associated hypothalamic–pituitary function.

## Figures and Tables

**Table 1:** Comparison of abnormal and normal pituitary imaging of ECD subjects. *p* values listed for normal vs. abnormal pituitary imaging.

Variable	Study Cohort (ECD) (*n* = 61)	Abnormal Pituitary Imaging (*n* = 29)	Normal Pituitary Imaging (*n* = 32)	*p* Value
**Age, mean (SD), years**	54.3 (10.9)	50.6 (11.7)	57.7 (9.0)	0.010
**Sex, Female No. (%)**	15 (24.6)	7 (46.7%)	8 (53.3%)	1.0
**BMI, median (IQR)**	27.8 (24.8–32.9)	27.8 (24.7–33.3)	28.4 (25.3–32.7)	0.69
***BRAF*** **pathogenic variant, positive No. (%)/*n***	31 (54.4)/57	16 (51.6%)	15 (48.4%)	0.60
**Panhypopituitarism No. (%)**	9 (14.8%)	9 (100.0%)	0	<0.001
**Diabetes insipidus No. (%)**	22 (36.1%)	18 (81.8%)	4 (18.2%)	<0.001
**Central Hypogonadism**	29 (47.54%)	19 (65.52%)	13 (40.63%)	0.073
**Central Adrenal Insufficiency**	13 (21.3%)	6 (24.14%)	6 (18.75%)	0.76
**Central Hypothyroidism**	6 (9.84%)	5 (17.24%)	1 (3.13%)	0.09
**hsCRP, median (IQR)/*n*, mg/L**	12.2 (3.1–45.4)	13.3 (3.3–54.3)	6.9 (3.1–45.4)	0.82
**ESR, median (IQR)/*n*, mm/h**	23 (11.0–38.0)	28 (12.0–49.0)	21 (9.5–26.5)	0.079
**Number of deficits, median (IQR)**	1.0 (0–2.0)	2.0 (1.0–3.0)	1.0 (0–1.0)	0.004

**Table 2:** Pituitary imaging findings and their frequencies. Cumulative number of subjects with imaging abnormalities do not match the study number (*n* = 61) because several subjects had more than one imaging abnormality while others had normal pituitary imaging.

Imaging Abnormality	Number of Subjects
**Thickened pituitary stalk**	15 (24.6%)
**Abnormal enhancement**	11 (18.0%)
**Suprasellar mass**	2 (3.3%)
**Loss of the posterior pituitary bright spot**	22 (36.1%)
**Small pituitary**	9 (14.8%)
**Stalk deviation**	7 (11.5%)
**Pituitary encasement**	3 (4.9%)
**Empty sella**	
**Complete**	3 (4.9%)
**Partial**	4 (6.6%)
**Microadenoma**	1 (1.6%)
**Pars Intermedia cyst (incidental finding)**	3 (4.9%)
**Rathke’s cyst (incidental finding)**	1 (1.6%)

## Data Availability

Data are contained within the article and supplementary material. The remaining data presented in this study are available on request from the corresponding author.

## Supplementary Materials

The following are available online at https://www.mdpi.com/article/10.3390/cancers13164126/s1, Supplement 1: Hormone deficits, treatment, and pituitary imaging characteristics of ECD cohort, Supplement 2: T1 weighted MRI scans of patients with pituitary imaging abnormalities. A represents coronal images, B represents sagittal images. Red arrows point to the pituitary gland structures of interest.
