# Supplementary material for: Pituitary Imaging Abnormalities and Related Endocrine Disorders in Erdheim–Chester Disease
Source: Cancers (Basel). 2021 Aug 17;13(16):4126. doi: 10.3390/cancers13164126 (PMC8392147; doi:10.3390/cancers13164126)
Supplement: Supplementary file 1 [file cancers-13-04126-s001.zip › Supplement 1.pdf]

Supplementary Materials

Pituitary Imaging Abnormalities and Related Endocrine Disorders in Erdheim–Chester Disease

Skand Shekhar, Jorge A. Irizarry-Caro, Ninet Sinaii, William A. Gahl, Juvianee I. Estrada-Veras, Rahul H. Dave, Bernadette R. Gochuico, Georgios Z. Papadakis, Nicholas Patronas, Constantine A. Stratakis, Kevin O'Brien and Fady Hannah-Shmouni

Supplement 1 (Table). Hormone deficits, treatment and pituitary imaging characteristics of ECD cohort.

ECD Serial No/ Sex	Age at NIH Visit	Age at Diagnosis of ECD	Endocrine Abnormalities	Hypothalamic-Pituitary Lesion on MRI	Treatment for ECD
**1-M**	46	43	Central hypogonadism, DI	Small pituitary; narrowing of the pituitary stalk; loss of pituitary bright spot	Imatinib
**2-M**	42	42	Central AI, central hypogonadism, DI	Stalk deviation	PEG-IFN, steroids
**3-F**	32	32	None	Thickened pituitary stalk	PEG-IFN
**4-M**	64	54	Central hypogonadism	Abnormal enhancement; pituitary encasement; possible cyst intermediate; absent posterior lobe	Anakinra
**5-M**	47	43	Central hypogonadism	Partial empty sella	Interferon alfa
**6-M**	48	44	Central AI, central hypogonadism, central hypothyroidism, DI	Abnormal enhancement; absent posterior lobe; suprasellar mass causing lateral displacement of pituitary segments; loss of pituitary bright spot	Methotrexate, Remicade, Ionizing radiation
**7-M**	68	67	Central hypogonadism	Small pituitary; thickened pituitary stalk; tapering of the infundibulum; partially empty sella; loss of pituitary bright spot	Interferon alfa
**8-M**	42	36	Central hypogonadism, central hypothyroidism, DI	Enlarged pituitary stalk at hypothalamus; secondary empty sella with small pituitary; loss of pituitary bright spot	Anakinra, Interferon alfa
**9-M**	50	46	Central hypogonadism, DI	Thickened stalk at the base and right side of pituitary, invading the cavernous sinus; abnormal pituitary enhancement	Interferon alfa
**10-M**	64	63	None	None	None
**11-F**	46	44	DI	None	Cladribine
**12-M**	41	36	Central hypogonadism, DI	None	Dabrafenib, Trametinib, Interferon alfa
**13-M**	51	48	Central AI, central hypogonadism, central hypothyroidism, DI	Thickened stalk; pituitary encasement	Dabrafenib, Trametinib
**14-M**	64	57	DI	Loss of pituitary bright spot	Interferon alfa
**15-M**	51	46	None	Abnormal pituitary morphology; thickened pituitary stalk	Cladribine
**16-M**	57	58	Central AI	None	Vemurafenib
**17-M**	61	59	None	None	Imatinib, Dabrafenib, Trametinib
**18-F**	32	28	DI	Loss of pituitary bright spot	Ionizing radiation, Cladribine, Interferon alfa
**19-M**	57	57	None	Loss of pituitary bright spot	None
**20-F**	19	17	None	Abnormal pituitary morphology; thickened pituitary stalk	PEG-IFN

**21-F**	64	64	None	Small pituitary	Cladribine
**22-F**	62	56	Central hypothyroid-ism	Small pituitary; partially empty sella	Dabrafenib, Trametinib
**23-F**	50	50	Central AI, central hy-pogonadism, hy-perprolactinemia	Cyst intermediate	6-Mercaptopurine, Vin-cristine
**24-M**	67	61	None	Small pituitary; partially empty sella	Anakinra
**25-F**	57	53	None	Cyst intermediate	Cladribine
**26-M**	39	38	Central AI, central hy-pogonadism, DI, hy-perprolactinemia	Small pituitary; thickened pituitary stalk; loss of pituitary bright spot	Interferon alfa
**27-M**	34	33	Central AI, central hy-pogonadism, central hypothyroidism, DI	Abnormal pituitary morphology-hetero-genous enhancement; small pituitary; thickened pituitary stalk; stalk deviation; microadenoma; loss of pituitary bright spot	None
**28-M**	51	51	Central hypogonad-ism, hyperprolac-tinemia	None	Interferon alfa
**29-M**	45	42	None	None	None
**30-M**	50	49	None	None	PEG-IFN
**31-F**	48	47	Central hypogonad-ism, DI, hyperprolac-tinemia	Cyst intermediate; suprasellar mass; loss of pituitary bright spot	Interferon alfa
**35-M**	45	44	Central hypogonad-ism, DI	Heterogenous enhancement of pituitary gland; small pituitary; thickened pituitary stalk; partially empty sella; loss of pitui-tary bright spot	Vemurafenib
**36-M**	61	61	Central hypogonadism	None	6-Mercaptopurine, Ster-oids, Vinblastine
**37-M**	72	69	DI	None	Anakinra
**38-M**	54	52	Central hypogonad-ism, DI	None	Interferon alfa, Dabraf-enib, Trametinib
**39-M**	56	51	None	None	Vemurafenib
**40-M**	59	58	Central hypogonadism	None	Vemurafenib
**41-M**	37	37	None	None	Anakinra
**42-M**	58	57	Central hypogonad-ism, DI	None	Interferon alfa
**43-M**	55	51	Central AI, central hy-pogonadism, DI	Loss of pituitary bright spot	Vemurafenib
**44-M**	68	65	None	None	Cyclophosphamide, Steroids
**45-F**	30	28	Central AI, central hy-pogonadism, central hypothyroidism, DI, hyperprolactinemia	Thickened pituitary stalk; pituitary gland enlarged abutting the optic chiasm; loss of pituitary bright spot	Cladribine
**46-M**	47	39	Central AI	Pituitary was symmetrical	Remicade
**47-M**	44	34	DI	Loss of pituitary bright spot	Interferon alfa, dabraf-enib, trametinib
**48-M**	61	61	Central AI, central hy-pogonadism	Abnormal configuration of the pituitary; thickened pituitary stalk; stalk deviation; pituitary encasement; partially empty sella; loss of pituitary bright spot	Steroids
**49-M**	56	56	Central AI, central hy-pogonadism	None	None

**50-M**	45	42	Central hypogonadism, DI	Thickened pituitary stalk	Dasatinib
**51-F**	64	63	None	Thickened pituitary stalk; stalk deviation; loss of pituitary bright spot	Anakinra
**52-F**	48	48	Central hypogonadism, hyperprolactinemia	None	PEG-IFN
**53-M**	65	65	Central hypogonadism, hyperprolactinemia	Rathke's cyst	None
**54-M**	64	61	Central hypogonadism, DI	Abnormal pituitary morphology-heterogenous enhancement; loss of pituitary bright spot	Vemurafenib
**55-M**	58	58	Central hypogonadism, DI	Loss of pituitary bright spot	Interferon alfa
**56-F**	64	61	None	None	Anakinra, Vemurafenib
**57-M**	50	46	Central hypogonadism, DI	Thickened pituitary stalk; loss of pituitary bright spot	None
**58-M**	52	52	None	Stalk deviation	Vemurafenib
**59-F**	58	58	None	Thickened pituitary stalk; stalk deviation; loss of pituitary bright spot	None
**60-F**	57	47	Central AI, hyperprolactinemia	None	Interferon alfa
**64-M**	69	68	None	None	Methotrexate
**67-M**	47	47	None	Thickened pituitary stalk; stalk deviation; loss of pituitary bright spot	Dabrafenib, Trametinib
**68-M**	74	74	Central AI, central hypogonadism, hyperprolactinemia	Loss of pituitary bright spot	Steroids
**71-M**	46	45	Central hypogonadism	None	Dabrafenib, Trametinib

AI, adrenal insufficiency; DI, diabetes insipidus; PEG-IFN, pegylated interferon.
