# Supplementary material for: Pituitary Imaging Abnormalities and Related Endocrine Disorders in Erdheim–Chester Disease
Source: Cancers (Basel). 2021 Aug 17;13(16):4126. doi: 10.3390/cancers13164126 (PMC8392147; doi:10.3390/cancers13164126)
Supplement: Supplementary file 1 [file cancers-13-04126-s001.zip › Supplement 2.pptx]

## Slide 1
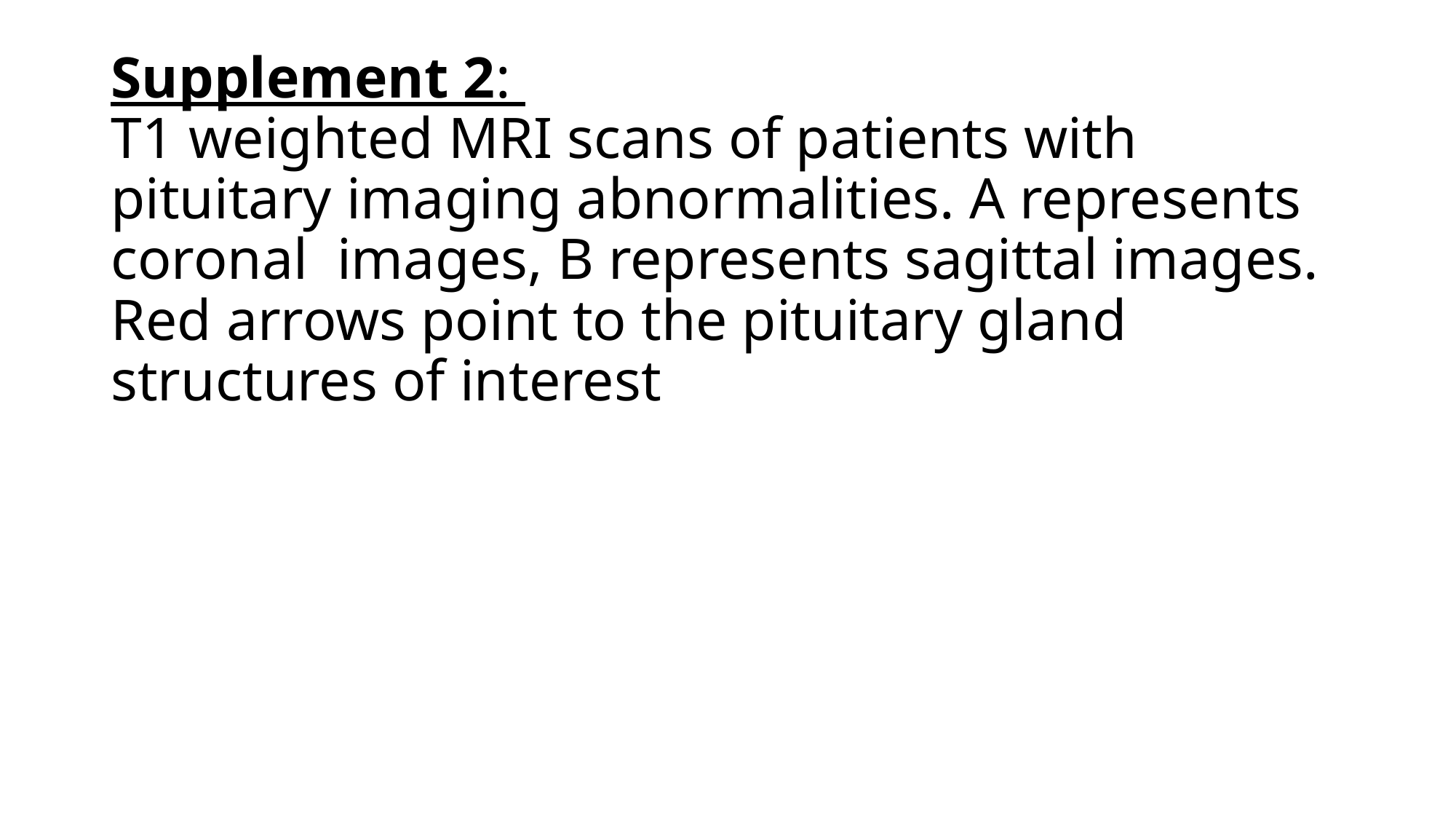

# Supplement 2: T1 weighted MRI scans of patients with pituitary imaging abnormalities. A represents coronal images, B represents sagittal images. Red arrows point to the pituitary gland structures of interest

## Slide 2
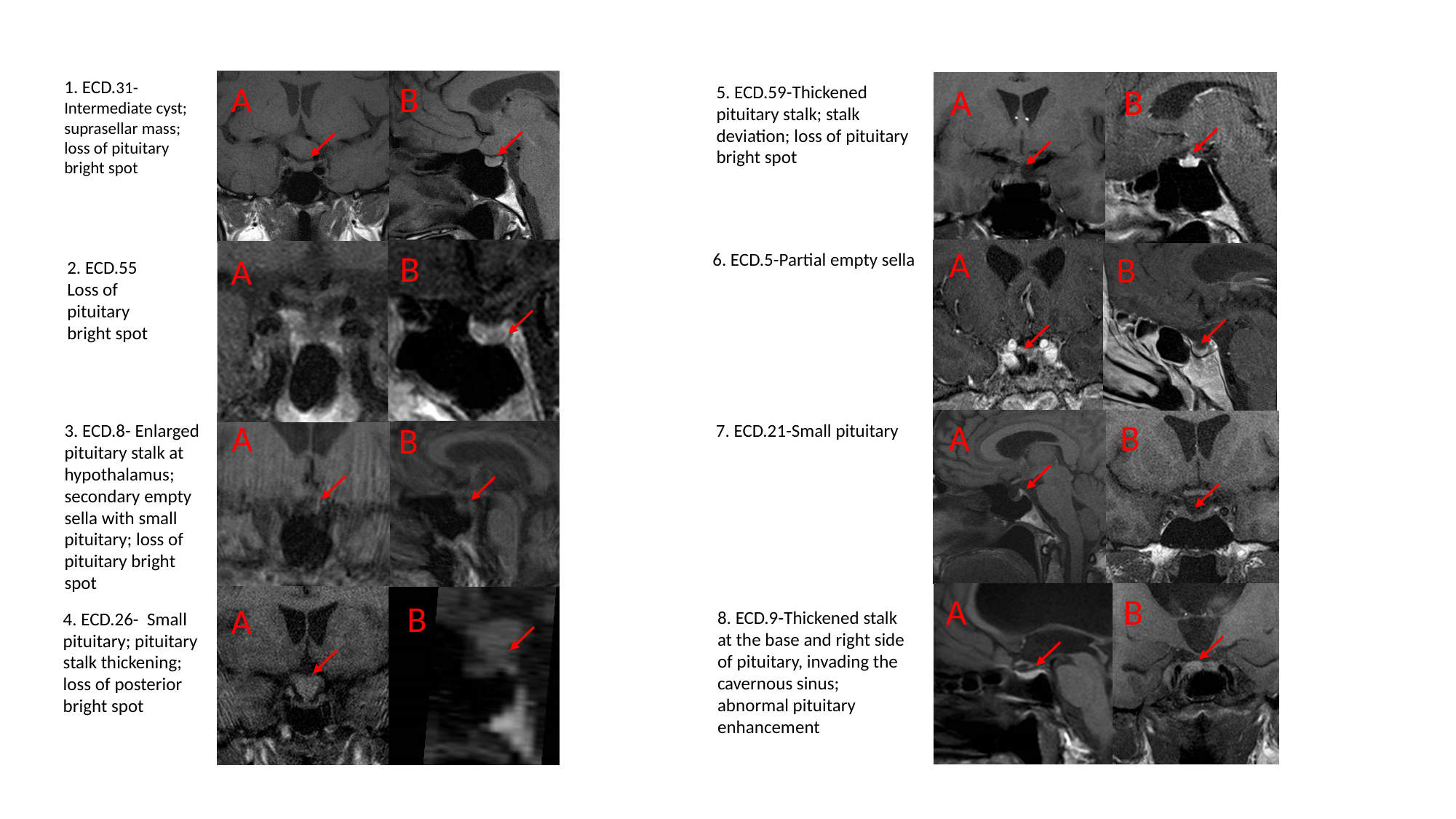

1. ECD.31-Intermediate cyst; suprasellar mass; loss of pituitary bright spot
A
B
A
B
5. ECD.59-Thickened pituitary stalk; stalk deviation; loss of pituitary bright spot
A
B
B
6. ECD.5-Partial empty sella
A
2. ECD.55 Loss of pituitary bright spot
B
A
A
B
3. ECD.8- Enlarged pituitary stalk at hypothalamus; secondary empty sella with small pituitary; loss of pituitary bright spot
7. ECD.21-Small pituitary
B
A
B
A
8. ECD.9-Thickened stalk at the base and right side of pituitary, invading the cavernous sinus; abnormal pituitary enhancement
4. ECD.26- Small pituitary; pituitary stalk thickening; loss of posterior bright spot

## Slide 3
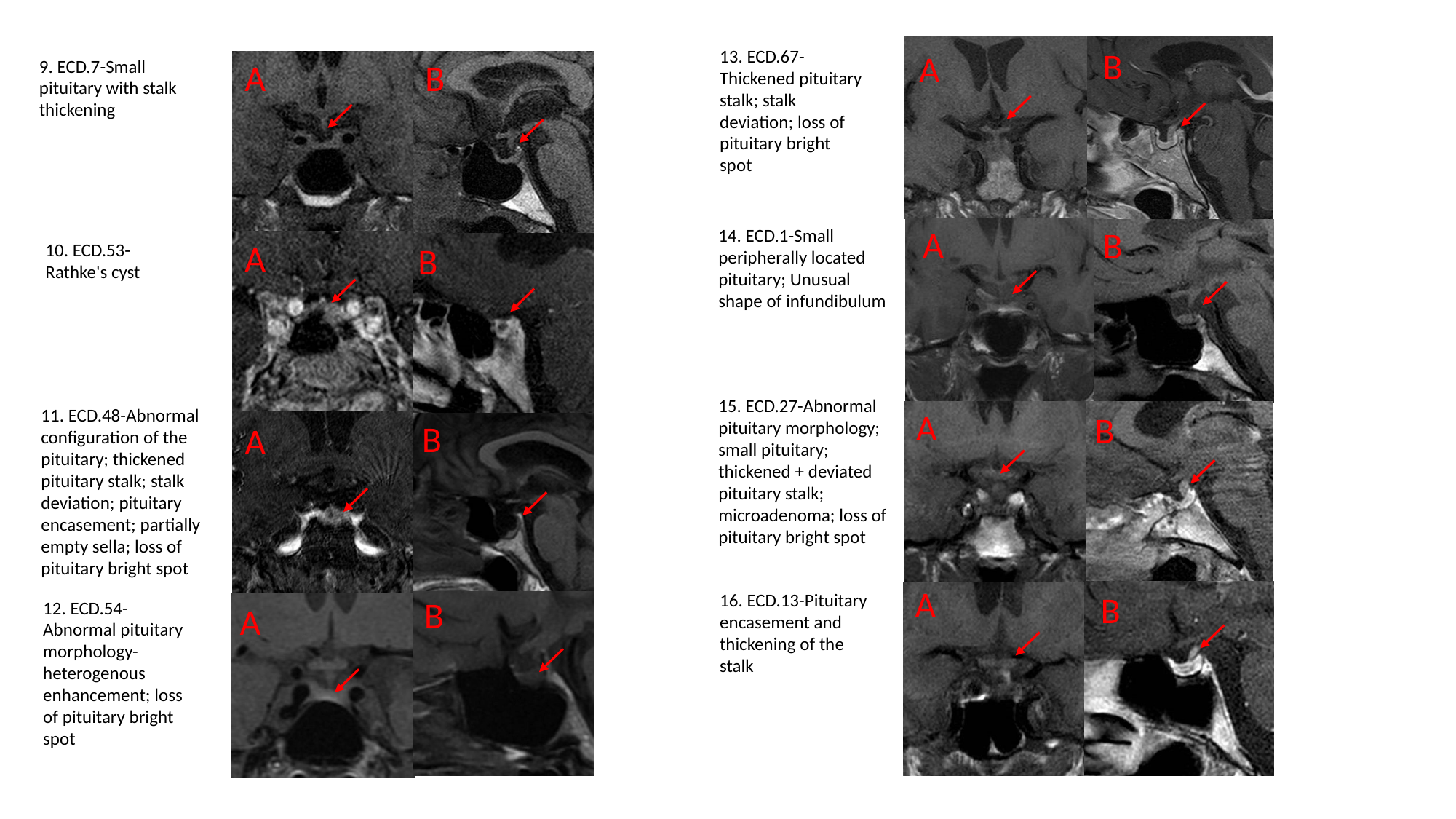

B
13. ECD.67-Thickened pituitary stalk; stalk deviation; loss of pituitary bright spot
A
9. ECD.7-Small pituitary with stalk thickening
A
B
A
B
14. ECD.1-Small peripherally located pituitary; Unusual shape of infundibulum
A
B
10. ECD.53-Rathke's cyst
15. ECD.27-Abnormal pituitary morphology; small pituitary; thickened + deviated pituitary stalk; microadenoma; loss of pituitary bright spot
11. ECD.48-Abnormal configuration of the pituitary; thickened pituitary stalk; stalk deviation; pituitary encasement; partially empty sella; loss of pituitary bright spot
A
B
B
A
A
B
16. ECD.13-Pituitary encasement and thickening of the stalk
B
12. ECD.54-Abnormal pituitary morphology-heterogenous enhancement; loss of pituitary bright spot
A

## Slide 4
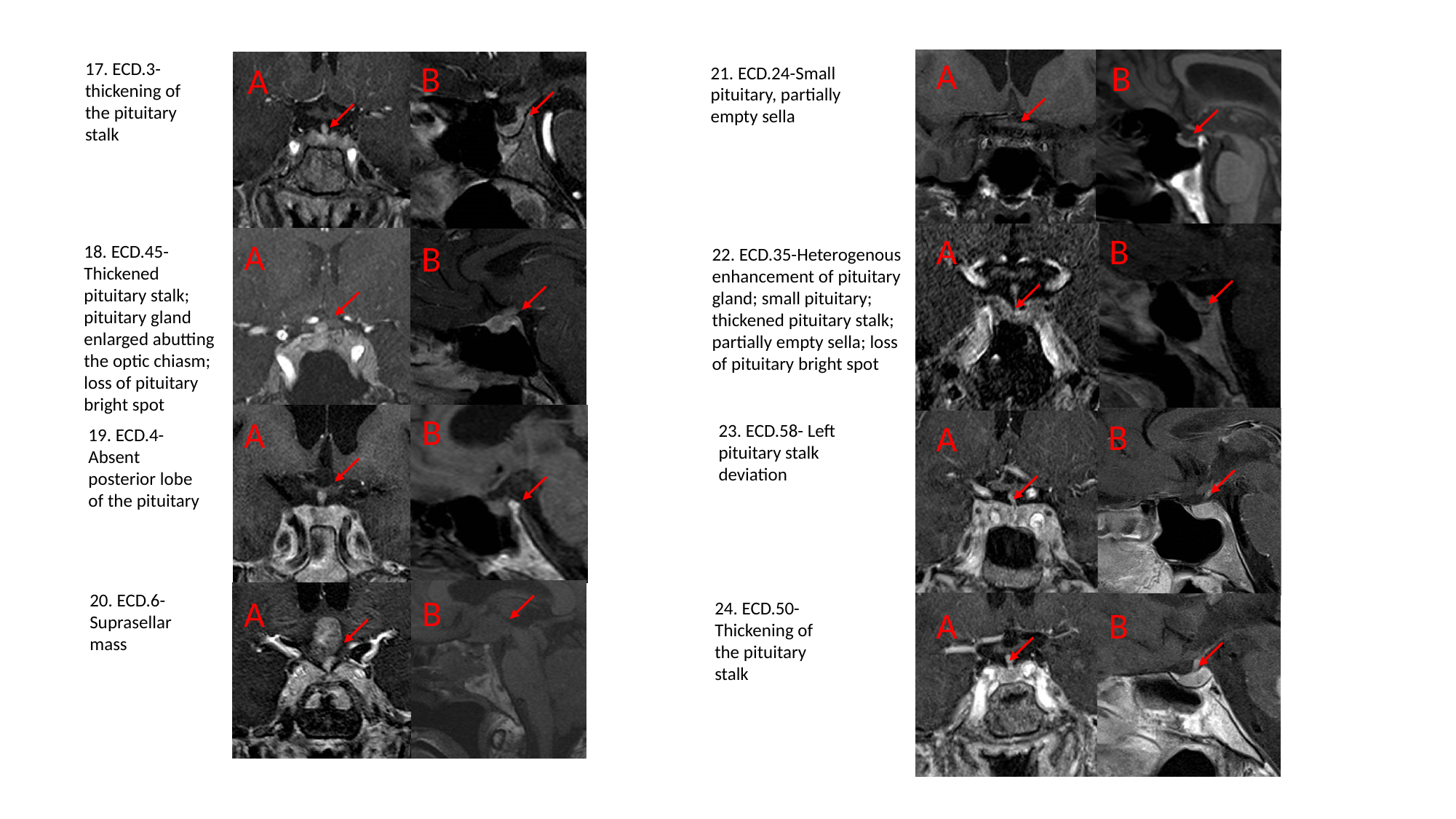

A
B
B
17. ECD.3- thickening of the pituitary stalk
A
21. ECD.24-Small pituitary, partially empty sella
A
B
A
B
18. ECD.45-Thickened pituitary stalk; pituitary gland enlarged abutting the optic chiasm; loss of pituitary bright spot
22. ECD.35-Heterogenous enhancement of pituitary gland; small pituitary; thickened pituitary stalk; partially empty sella; loss of pituitary bright spot
B
A
B
A
23. ECD.58- Left pituitary stalk deviation
19. ECD.4- Absent posterior lobe of the pituitary
20. ECD.6-Suprasellar mass
B
A
24. ECD.50- Thickening of the pituitary stalk
A
B

## Slide 5
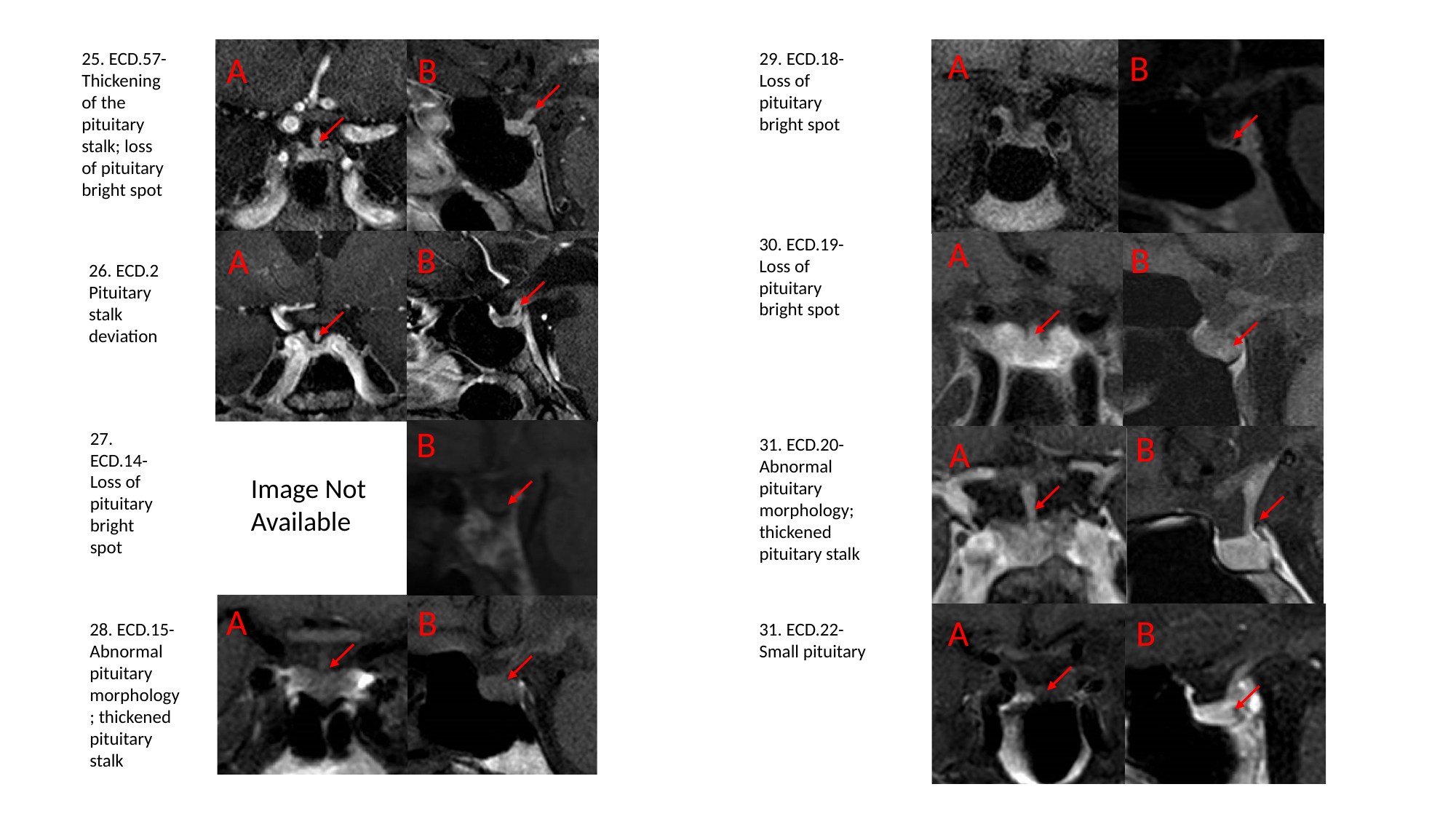

A
B
25. ECD.57- Thickening of the pituitary stalk; loss of pituitary bright spot
A
B
29. ECD.18- Loss of pituitary bright spot
A
30. ECD.19- Loss of pituitary bright spot
B
B
A
26. ECD.2 Pituitary stalk deviation
B
B
27. ECD.14- Loss of pituitary bright spot
A
31. ECD.20- Abnormal pituitary morphology; thickened pituitary stalk
Image Not
Available
A
B
B
A
28. ECD.15- Abnormal pituitary morphology; thickened pituitary stalk
31. ECD.22- Small pituitary

## Slide 6
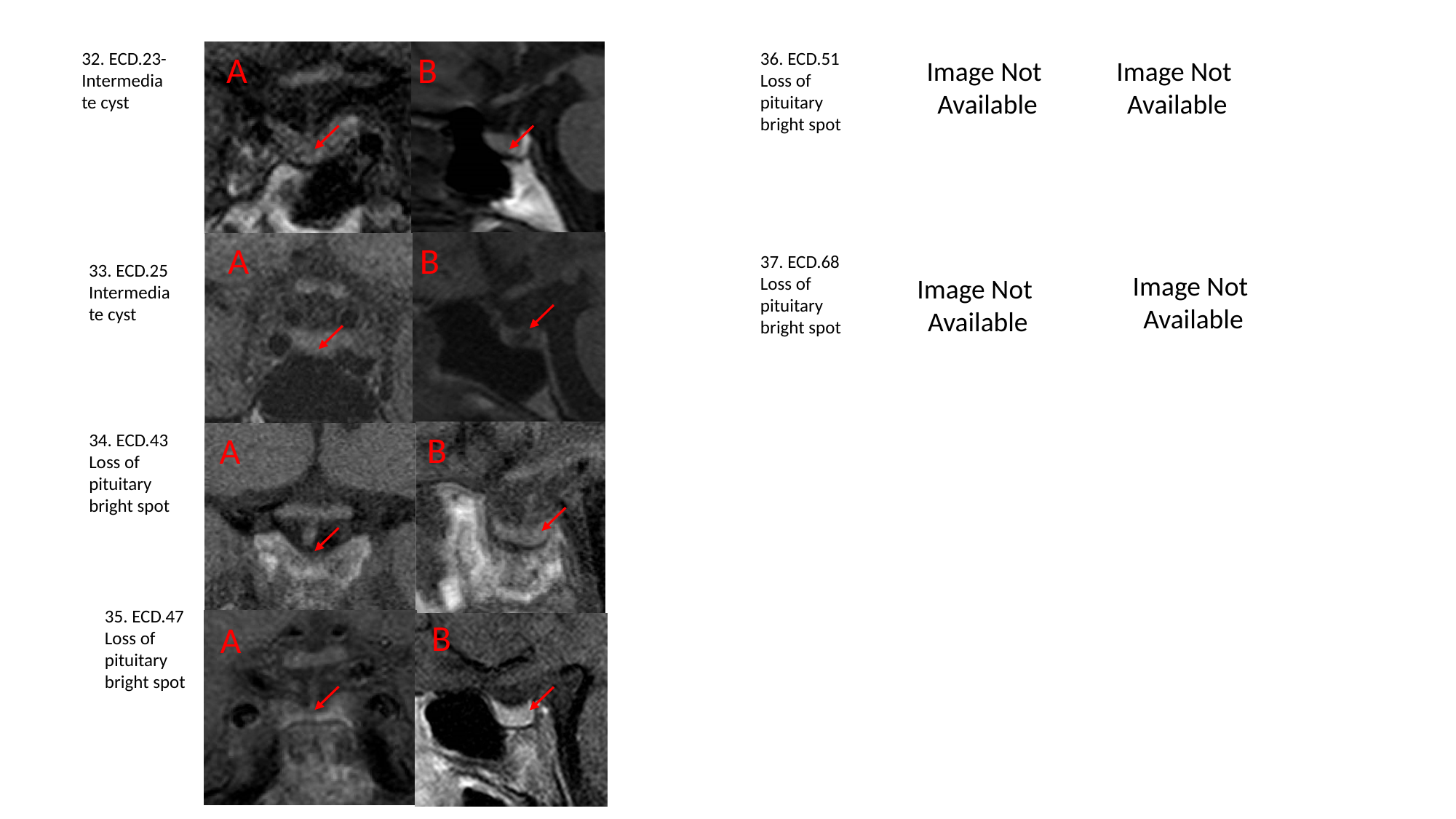

32. ECD.23-Intermediate cyst
A
B
36. ECD.51 Loss of pituitary bright spot
Image Not
Available
Image Not
Available
A
B
37. ECD.68 Loss of pituitary bright spot
33. ECD.25 Intermediate cyst
Image Not
Available
Image Not
Available
B
A
34. ECD.43 Loss of pituitary bright spot
35. ECD.47 Loss of pituitary bright spot
B
A
